# Pirfenidone inhibits TGF-β1-induced over-expression of collagen type I and heat shock protein 47 in A549 cells

**DOI:** 10.1186/1471-2466-12-24

**Published:** 2012-06-13

**Authors:** Keiko Hisatomi, Hiroshi Mukae, Noriho Sakamoto, Yuji Ishimatsu, Tomoyuki Kakugawa, Shintaro Hara, Hanako Fujita, Seiko Nakamichi, Hisashi Oku, Yoshishige Urata, Hiroshi Kubota, Kazuhiro Nagata, Shigeru Kohno

**Affiliations:** 1 Second Department of Internal Medicine, Nagasaki University School of Medicine, 1-7-1 Sakamoto, Nagasaki, 852-8501, Japan; 2 Department of Respiratory Medicine, University of Occupational and Environmental Health, Kitakyushu, Japan; 3 Department of General Medicine, Nagasaki University Hospital, Nagasaki, Japan; 4 Discovery Research Laboratories, Shionogi & Co. Ltd, Osaka, Japan; 5 Department of Stem Cell Biology, Nagasaki University Graduate of Biomedical Science, Nagasaki, Japan; 6 Department of Molecular and Cellular Biology, Institute for Frontier Medical Science, Kyoto University, Kyoto, Japan

**Keywords:** Pneumocyte, Interstitial pneumonia, Epithelial cell, Epithelial mesenchymal transition, Pulmonary fibrosis

## Abstract

**Background:**

Pirfenidone is a novel anti-fibrotic and anti-inflammatory agent that inhibits the progression of fibrosis in animal models and in patients with idiopathic pulmonary fibrosis (IPF). We previously showed that pirfenidone inhibits the over-expression of collagen type I and of heat shock protein (HSP) 47, a collagen-specific molecular chaperone, in human lung fibroblasts stimulated with transforming growth factor (TGF)-β1 *in vitro*. The increased numbers of HSP47-positive type II pneumocytes as well as fibroblasts were also diminished by pirfenidone in an animal model of pulmonary fibrosis induced by bleomycin. The present study evaluates the effects of pirfenidone on collagen type I and HSP47 expression in the human alveolar epithelial cell line, A549 cells *in vitro*.

**Methods:**

The expression of collagen type I, HSP47 and E-cadherin mRNAs in A549 cells stimulated with TGF-β1 was evaluated by Northern blotting or real-time PCR. The expression of collagen type I, HSP47 and fibronectin proteins was assessed by immunocytochemical staining.

**Results:**

TGF-β1 stimulated collagen type I and HSP47 mRNA and protein expression in A549 cells, and pirfenidone significantly inhibited this process. Pirfenidone also inhibited over-expression of the fibroblast phenotypic marker fibronectin in A549 cells induced by TGF-β1.

**Conclusion:**

We concluded that the anti-fibrotic effects of pirfenidone might be mediated not only through the direct inhibition of collagen type I expression but also through the inhibition of HSP47 expression in alveolar epithelial cells, which results in reduced collagen synthesis in lung fibrosis. Furthermore, pirfenidone might partially inhibit the epithelial-mesenchymal transition.

## Background

Idiopathic pulmonary fibrosis (IPF) is a progressive and fatal disorder characterized by patchy fibrotic areas with fibroblast proliferation and extracellular matrix remodeling that results in irreversible distortion of the lung architecture. The underlying molecular mechanisms through which excessive collagen is deposited in fibrotic lesions are not fully understood. The pathological features of IPF include scattered fibroblast foci that consist of aggregates of mesenchymal cells, and which are associated with the progression of fibrosis [1]. Although resident fibroblasts probably expand in response to injury, recent studies support the notion that lung fibroblast development might also be derived from bone marrow and/or epithelial cells [2]. The alveolar epithelial to mesenchymal transition (EMT) has been studied in detail from the viewpoint of the pathogenesis of lung, as well as renal fibrosis [2-5]. Alveolar epithelial cells are also the primary source of mediators such as platelet-derived growth factor, transforming growth factor (TGF)-β1, tumor necrosis factor (TNF)-α and connective tissue growth factor that can induce fibroblast migration, proliferation and activation [6]. Thus, alveolar epithelial cells as well as fibroblasts are important in the pathogenesis of lung fibrosis.

Pirfenidone is a novel antifibrotic agent that inhibits the progression of lung, kidney and hepatic fibrosis in experimental models [7-13]. This drug also exerts anti-inflammatory properties including the regulation of key growth factors and cytokines [10,11,14-16], and recent clinical trials have revealed its therapeutic effects in patients with IPF [17-19]. However, the exact mechanisms through which pirfenidone offers protection against lung fibrosis remain unclear. In this context, we recently found that pirfenidone inhibits the expression of collagen type I and heat shock protein (HSP) 47, a collagen-specific molecular chaperone, in TGF-β-stimulated human lung fibroblasts *in vitro*[20]. Using a mouse model of bleomycin-induced pulmonary fibrosis, we also discovered that pirfenidone inhibits HSP47 over-expression in myofibroblasts and in type II pneumocytes, especially in fibrotic lesions, and that this activity is concomitant with an obvious reduction in fibrosis [12]. HSP47 is localized in the endoplasmic reticulum that participates in the intracellular processing, folding, assembly and secretion of procollagens [21-23]. Irrespective of the tissue site and organ, HSP47 expression is always induced during the process of fibrosis, particularly in and around fibrotic lesions [12,24,25]. Therefore, HSP47-positive cells, especially myofibroblasts, might play a central role in the synthesis, deposition and remodeling of the ECM in pulmonary fibrosis in both patients and in animal models [12,26,27]. In this context, collagen type I and HSP47 expression is recently considered to be one of the useful parameters for recognizing EMT [28]. However, the specific effects of pirfenidone upon collagen and HSP47 synthesis and EMT in alveolar epithelial cells *in vitro* remain obscure.

To clarify the association between alveolar epithelial cells and lung fibrosis and the action of pirfenidone *in vitro*, we examined the expression of collagen type I and HSP47 in the human alveolar epithelial cell line, A549, stimulated with TGF-β1.

## Materials and methods

### Cells and Reagents

The human type II alveolar epithelial cell line, A549 (The American Type Culture Collection, Manassas, VA), was maintained in Dulbecco's modified minimal essential medium (DMEM) containing 10% FBS and 2 mM L-glutamine at 37 °C in a 5% CO_2_-humidified atmosphere. All experiments proceeded using cells after 3–5 cell passages. Pirfenidone was provided by Shionogi & Co., Ltd (Osaka, Japan). Recombinant human TGF-β1 (R&D Systems, Inc. Minneapolis, MN), was reconstituted in 4 mM HCl containing 0.1% bovine serum albumin (BSA) to prepare a 1 μg/ml stock solution of active growth factor. The stock solution was added to media to yield various final concentrations of TGF-β1. To control for nonspecific effects, cells were incubated in an equal volume of 4 mM HCl and BSA solution was added to the basal media.

### Incubation of cells with pirfenidone and TGF-β1

Subcultures of A549 cells were seeded in 60-mm culture dishes (BD Falcon^TM^, Franklin Lakes, NJ) at a density of 1 × 10^5^ per dish to analyze collagen type I and HSP47 mRNA expression and protein levels. When the cells reached 70 - 80% confluence, the medium was replaced with serum-free DMEM. The cells were subsequently stimulated for 3, 6, 12, 24, 48 and 72 h with culture medium alone (control) or with 100, 500 and 1000 μg/ml of pirfenidone with or without 0.1, 1, 5, 10 ng/ml of TGF-β1**.** Changes in cell morphology were also assessed under phase contrast light microscopy.

### RNA extraction and reverse-transcriptase PCR

Total RNA was isolated from the cells using the Qiagen RNeasy Mini Kit (Qiagen, Valencia, CA) according to the manufacturer's instructions. Samples were digested with DNase I (Qiagen) to remove contaminating genomic DNA. Isolated total RNA was analyzed using a DU 800 UV/Visible Spectrophotometer (Beckman Coulter, Fullerton, CA). To generate complementary DNA (cDNA), total RNA (5 μg) was reverse-transcribed using the SuperScript^TM^ III first strand synthesis kit (Invitrogen, Carlsbad, CA) with oligo deoxythymidine as a primer. The reverse transcriptase reaction proceeded in a total volume of 20 μl in a Gene Amp PCR system 9700 (Applied Biosystems, Foster City, CA) at 65 °C for 5 minutes, followed by 50 minutes at 42 °C and 15 minutes at 70 °C. Reaction volumes of 25 μl were placed in 96-well optical reaction plates with adhesive covers (ABI Prism^TM^ Applied Biosystems) using TaqMan^TM^ Universal PCR Master Mix (Applied Biosystems). Human beta-actin (β-actin) served as the internal control. TaqMan probes for human E-cadherin and β-actin were purchased from Applied Biosystems. Samples were heated to 95 °C for 10 min and then PCR amplification was achieved by 40 cycles at 95 °C for 15 s and at 60 °C for 1 min using the ABI Prism 7500 (Applied Biosystems) as described by the manufacturer. Real-time data were analyzed using the comparative C_T_ method, where C_T_ is the cycle number at which the fluorescence reading is first recorded above background levels. The comparative C_T_ and standard curve methods are similar except the former uses the arithmetic formula, 2^–ΔΔCT^ to achieve relative quantification. A prior validation experiment demonstrated that amplification efficiencies of the primer/probe sets of the target genes and β-actin were approximately equal and within 0.1.

### Northern blotting

Samples were Northern blotted for collagen type I and HSP47 as described [20,29]. Human HSP47 and collagen-type I complementary DNAs (cDNAs) were provided by the Institute for Frontier Medical Science, Kyoto University, Japan. HSP47 mRNA was detected using a 1.5 kilobase pair (kbp) *Eco*RI fragment of full-length human HSP47 (CBP2) cDNA as a probe, whereas collagen type I mRNA was detected using a 0.6 kbp *Eco*RV fragment of full-length pCOLA1-I-CP cDNA as a probe. The probes were labeled with ^32^P using a Random Primer Labeling kit (Takara Biomedicals, Shiga, Japan). Isolated RNAs (10 μg) were resolved by electrophoresis on 1% agarose gels containing 1.2% formaldehyde, transferred to a nylon membrane (Hybond^TM^-N^+^, Amersham International, Amersham, UK), and then separately hybridized with each ^32^P-labeled probe for 24 h at 42 °C. Autoradiographed membranes were assessed using a BAS5000 Bioimage Analyzer (Fuji Photo Film, Japan). Relative transcription was normalized against hybridization with a glyceraldehyde-3-phosphate dehydrogenase (GAPDH) probe.

### Immunocytochemistry

After a 48-h incubation as detailed above, cultured cells were fixed with acetone for 10 min and stained using the alkaline phosphatase anti-alkaline phosphatase method with goat anti-human collagen type I polyclonal (CHEMICON, Temecula, CA), mouse anti-human HSP47 (Stressgen, Victoria, Canada) and mouse anti-cellular fibronectin monoclonal (CHEMICON) antibodies. Negative controls included an irrelevant mouse or goat IgG (Santa Cruz Biotechnology, Santa Cruz, CA). The staining intensity of collagen type I and HSP47 in A549 cells was semi-quantified at × 400 magnification in five fields using a grading score of 0 to 3 (0, 1, 2 and 3, corresponding to absent, weak, moderate and intense staining, respectively). The total cell count was converted to 100, so the maximum number was 300 in this scoring system. The interobserver and intraobserver variability in assessing the results was reproducible. Representative results from one observer were statistically analyzed.

### Statistical analysis

All values are expressed as means ± SEM. The minimum number of replicates for all measurements was at least 3. Differences between multiple groups were compared by one-way analysis of variance. Dunnett's test served as the post hoc test for multiple comparisons. Significance was assumed at *p* < 0.05.

## Results

### TGF-β1 enhances the expression of collagen type I and HSP47 mRNA and protein in A549 cells

**Figure 1:**
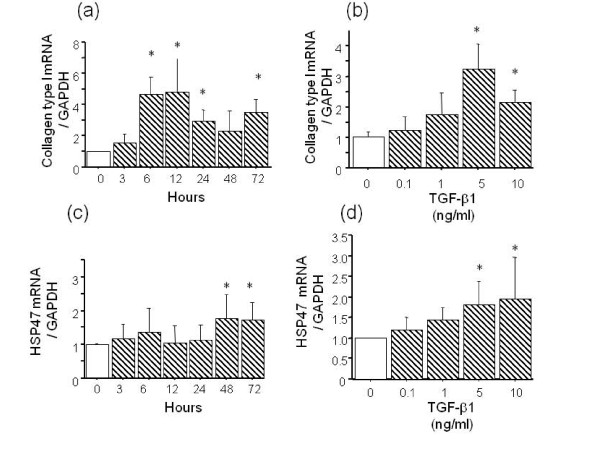
**Time course of collagen type I (a) and HSP47 (c) mRNA expression in A549 cells incubated with 5 ng/ml of TGF-β1 analyzed by Northern blotting.** Collagen type I (**b**) and HSP47 (**d**) mRNA expression was significantly increased in A549 cells after 6 and 48 h incubation with 5 and 10 ng/ml of TGF-β1, respectively. Density of mRNA and GAPDH bands was compared and results are shown as ratios. Values are means ± SEM of four experiments. *P < 0.05, compared with control.

**Figure 2:**
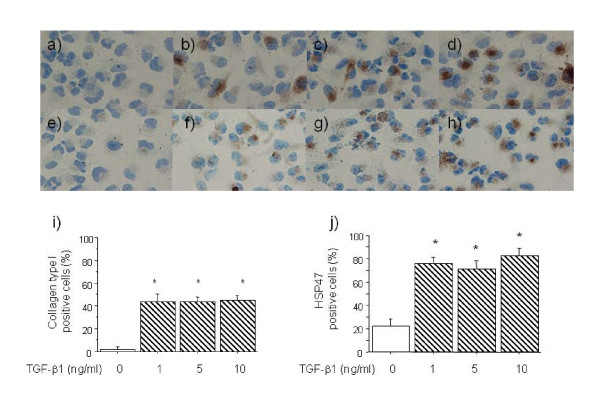
**Immunocytochemistry findings of collagen type I and HSP47 expression in A549 cells incubated for 48 h with various concentrations of TGF-β1.** Collagen expression: 0 (**a**), 1 (**b**), 5 (**c**), and 10 ng/ml (**d**) of TGF-β1. HSP47 expression: 0 (**e**), 1 (**f**), 5 (**g**), and 10 ng/ml (**h**) of TGF-β1. Original magnification, x400. Positive rates of collagen type I (**i**) and HSP47 (**j**) immunostaining of A549 cells after incubation with TGF-β1 (0, 1, 5, 10 ng/ml) for 48 h. Positive rate of stained cells are significantly increased by TGF-β1 compared with control. Values are means ± SEM of 5 experiments.

We first determined the time course of the TGF-β1 (5 ng/ml) effect on the mRNA expression of collagen type I and HSP47 in A549 cells. The response of collagen type I to 5 ng/ml of TGF-β1 was maximal after 6, 12, 24 and 72 h of incubation (Figure 1a). TGF-β1 at 5 and 10 ng/ml also significantly increased the expression of collagen type I mRNA after 6 h of incubation (Figure 1b). The response of HSP47 to 5 ng/ml TGF-β1 was maximal after 48 and 72 h of incubation (Figure 1c). Figure 1d shows that TGF-β1 at 5 and 10 ng/ml significantly increased HSP47 mRNA expression compared with untreated controls after 48 h. We next examined the effects of TGF-β1 on collagen type I and HSP47 protein expression in A549 cells using immunocytochemical staining. We found that 1, 5 and 10 ng/ml of TGF-β1 increased the number of cells that were immunopositive for both collagen type I and HSP47 at 48 h (Figure 2).

### Effects of pirfenidone on production of collagen type I and HSP47 in TGF-β1-stimulated A549 cells

**Figure 3:**
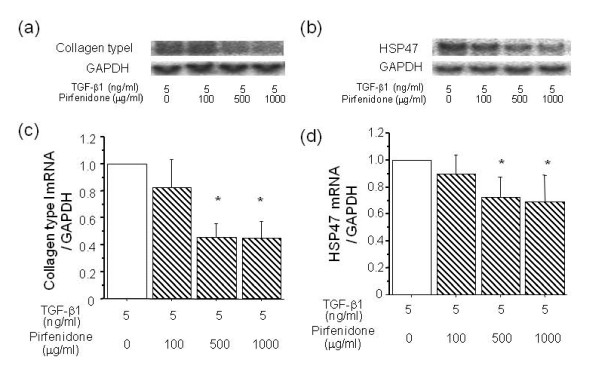
**Effects of pirfenidone on collagen type I and HSP47 mRNA expression in A549 cells.** Northern blots show that mRNA expression of collagen type I (**a**) and HSP47 (**b**) was inhibited in A549 cells after 6 and 48 h of incubation, with 500 and 1000 μg/ml, respectively, of pirfenidone and 5 ng/ml of TGF-β1. Density of mRNA bands was normalized against GAPDH mRNA band and is shown as ratios (**c** and **d**). Values are means ± SEM of 4 experiments.

**Figure 4:**
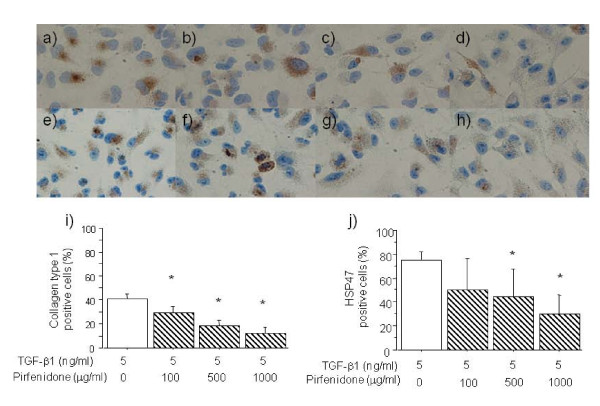
**Immunocytochemistry of collagen type I and HSP47 expression in A549 cells stimulated with TGF-β1 (5 ng/ml) and 0 (a, e), 100 (b, f), 500 (c, g), and 1000 μg/ml (d, h) of pirfenidone, respectively for 48 h.** Original magnification: x400. Positive rates for collagen type I (**i**) and HSP47 (**j**) immunostained A549 cells were significantly inhibited by 100, 500, 1000 μg/ml and by 500 and 1000 μg/ml of pirfenidone, respectively. Values are means ± SEM of 5 experiments.

The effect of pirfenidone on TGF-β1-induced expression of collagen type I and HSP47 mRNA in A549 cells was analysed by Northern blotting (Figure 3). Incubation with 500 and 1000 μg/ml of pirfenidone induced significant downregulation of collagen type I (6 h) and of HSP47 (48 h) mRNA expression in A549 cells stimulated by TGF-β1 at 5 ng/ml. Figure 4 shows the immunocytochemical findings obtained using the anti-human collagen type I and HSP47 antibodies. Pirfenidone significantly decreased the numbers of collagen type I- and HSP47-immunopositive cells at 100, 500 and 1000 μg/ml, and at 500 and 1000 μg/ml, respectively.

### Effects of pirfenidone on EMT in TGF-β1-stimulated A549 cells

**Figure 5:**
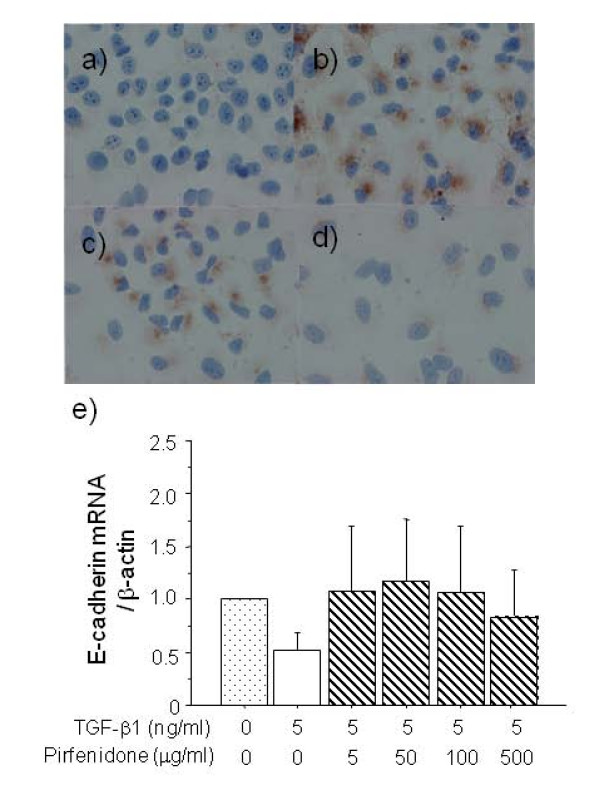
**Immunocytochemistry of fibronectin expression in A549 cells stimulated without (a) or with 5 ng/ml (b, c, d) of TGF-β1 without (b), or with 100 (c) or 500 μg/ml (d) of pirfenidone for 48 h.** Original magnification: x400. Pirfenidone decreased fibronectin over-expression induced by TGF-β1. Loss of epithelial marker E-cadherin mRNA in A549 cells caused by TGF-β1 (5 ng/ml) was also normalized by pirfenidone, but the difference did not reach significance (**e**).

**Figure 6:**
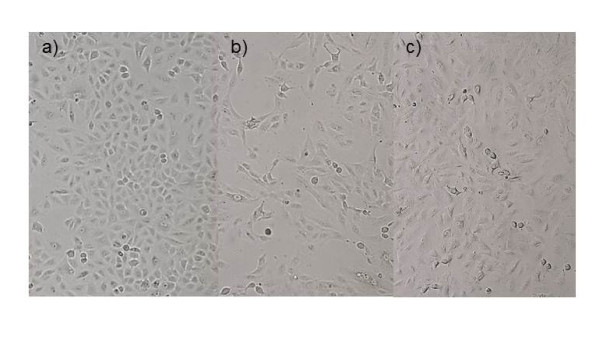
**Effects of pirfenidone on morphological changes stimulated by TGF-β1 for 48 h in A549 cells.** (**a**) Untreated A549 cells show a pebble-like epithelial shape (a) and TGF-β1-treated cells show a spindle-like mesenchymal shape (**b**). Pirfenidone partially suppresses TGF-β1-induced mesenchymal morphology (**c**).

Immunocytochemical staining of A549 cells showed that pirfenidone inhibited the TGF-β1-induced over-expression of fibronectin, which is a mesenchymal phenotypic marker (Figure 5a-d). Loss of the epithelial marker E-cadherin mRNA in A549 cells induced by TGF-β1 was also normalized by pirfenidone, but this difference did not reach significance (Figure 5e). In addition to the changes in the phenotypic markers, changes in cell morphology were also assessed under phase contrast light microscopy. Untreated A549 cells show a pebble-like epithelial shape and TGF-β1-treated cells show a spindle-like mesenchymal shape. Pirfenidone partially suppressed TGF-β1-induced mesenchymal morphology (Figure 6).

## Discussion

The major finding of the present study was that TGF-β1 enhanced HSP47 and collagen type I synthesis in A549 cells at both the mRNA and protein levels and that pirfenidone directly inhibited these effects.

The collagen-binding, stress-inducible protein, HSP47, acts as a collagen-specific molecular chaperone in the intracellular processing of procollagen [21-23]. In a murine model of bleomycin-induced pulmonary lung fibrosis, HSP47 mRNA and protein expression predominantly increased in α-SMA-positive myofibroblasts of the lung interstitium, and the relative amounts of HSP47 mRNA in the lung significantly correlated with the hydroxyproline content [12,27]. In addition to fibroblasts, type II pneumocytes in this mouse model expressed HSP47, suggesting that type II pneumocytes also contribute to lung fibrosis [12]. The expression of type I procollagen and HSP47 was also significantly higher in type II pneumocytes identified in surgical biopsy specimens from patients with idiopathic usual interstitial pneumonia (UIP) than in those from patients with collagen vascular disease-associated UIP and idiopathic nonspecific interstitial pneumonia [26], which have better prognoses than idiopathic UIP [30]. These findings suggest that in addition to fibroblasts, type II pneumocytes also directly contribute to lung fibrosis thorough collagen and HSP47 synthesis.

Pirfenidone exerts both anti-inflammatory and anti-fibrotic activities in animal models of pulmonary fibrosis induced by bleomycin [9-12] and cyclophosphamide [13]. These studies *in vivo* showed that pirfenidone obviously reduced the histological and biochemical signs of lung fibrosis including the hydroxyproline content. Pirfenidone also decreased the levels of pulmonary protein and gene expression of some fibrogenic cytokines such as TGF-β1 [10] and platelet-derived growth factor (PDGF) [14]. Our recent study *in vitro* showed that pirfenidone directly inhibits collagen type I and HSP47 expression in TGF-β1-stimulated human lung fibroblasts [20]. Together, these findings indicate the potential of pirfenidone as a novel, broad-spectrum anti-fibrotic agent. In addition, pirfenidone suppresses the increased expression of HSP47 in our mouse model of lung fibrosis [12], suggesting that this drug could change the fibroblast phenotype. Here, we demonstrated a similar inhibitory effect of pirfenidone on collagen type I and HSP47 in A549 cells at both the protein and mRNA levels *in vitro*, confirming that the effect of pirfenidone on collagen and HSP47 in *vivo* represented a direct effect on type II pneumocytes. In addition to its anti-inflammatory action, pirfenidone might act as an anti-fibrotic agent for patients with IPF by directly inhibiting HSP47 and collagen production in type II pneumocytes as well as in fibroblasts in the human lung. In this context, our immunocytochemical study of collagen I showed that pirfenidone co-administration significantly reduced the ratio (%) of positive cells at 100 μg/ml and significantly inhibited TGF-β1-enhanced HSP47 and collagen type I mRNA expression at 500 μg/ml. This suggests that pirfenidone acts as an anti-fibrotic agent by directly inhibiting both HSP47 and collagen type I mRNA expression, which results in reduced collagen synthesis in type II pneumocytes. In support of this proposal, HSP47 inhibition by antisense oligodeoxynucleotides obviously suppresses collagen production in 3 T6 cells [23], experimental proliferative glomerulonephritis [31] and in peritoneal fibrosis [32], indicating that HSP47 is a promising target for the treatment of fibrotic diseases including IPF. This study thus identified pirfenidone as the first known agent that can control HSP47 and collagen expression in type II pneumocytes.

Recent evidence from studies of pulmonary fibrosis supports the role of EMT in the development of fibroblastic foci in IPF [2]. Yao and colleagues [33] showed that TGF-β1 induces EMT in alveolar epithelial cells from SD rats. Kasai and colleagues also reported that TGF-β1, but not TNF-α or interleukin-1β, induced A549 cells to undergo EMT [3]. Willis and colleagues also demonstrated that cultures of primary rat alveolar epithelial cells and a rat alveolar epithelial cell line undergo EMT in response to TGF-β1 stimulation [5]. Kim et al. also reported that the main source of mesenchymal expansion is lung epithelial cells from a mouse model of pulmonary fibrosis *in vivo*[4]. The induction of EMT is characterized by the expression of α-SMA, transformation of myofibroblast morphology, the increased formation of stress fibers by F-actin reorganization, and loss of the epithelial marker E-cadherin. Collagen type I and HSP47 expression is also considered to be one of the useful parameters for recognizing EMT [28]. These recent findings and our present data demonstrate that EMT develops in alveolar epithelial cells mediated by TGF-β1, suggesting that such development represents an important mechanism of myofibroblast production during pulmonary fibrosis. In addition, the present data showed that the over-expression of collagen type I, HSP47 and fibronectin induced by TGF-β1 in A549 cells was inhibited by pirfenidone. TGF-β1-induced loss of E-cadherin in A549 cells was also normalized by pirfenidone while this difference was not significant. In addition, pirfenidone inhibited a mesenchymal morphology induced by TGF-β1. These suggest that pirfenidone might partially inhibit EMT.

## Conclusion

The anti-fibrotic effects of pirfenidone might be mediated not only through the direct inhibition of collagen type I expression but also through the inhibition of HSP47 expression in alveolar epithelial cells, which results in reduced collagen synthesis in lung fibrosis. Furthermore, pirfenidone might partially inhibit the epithelial-mesenchymal transition.

## Competing interests

The authors alone are responsible for the content and writing of the paper. HO is an employee of Shionogi & Co.,Ltd. SK received manuscript fee and consultant from Shionogi & Co.,Ltd.

## Authors’ contributions

KH wrote manuscript and did laboratory work, TK, SH, HF, SN, and YU did laboratory work, NS, YI, HO, HK, and KN did study design and assisted manuscript-writing, HM, SK supervised all the experiment and manuscript-writing. All authors read and approved the final manuscript.

## Pre-publication history

The pre-publication history for this paper can be accessed here:

http://www.biomedcentral.com/1471-2466/12/24/prepub
